# Mechanism Analysis of OsZF8-Mediated Regulation of Rice Resistance to Sheath Blight

**DOI:** 10.3390/ijms25115787

**Published:** 2024-05-26

**Authors:** Yan Wang, Haining Wang, Liangkun Zhang, Yiming Wang, Songhong Wei, Lili Wang

**Affiliations:** 1 College of Plant Protection, Department of Plant Pathology, Shenyang Agricultural University, Shenyang 110866, China; wy8806@syau.edu.cn (Y.W.); 2023620001@syau.edu.cn (H.W.); 2022240648@stu.syau.edu.cn (L.Z.); 2023220524@stu.syau.edu.cn (Y.W.); 2 Liaoning Academy of Agricultural Sciences, Shenyang 110101, China

**Keywords:** *Rhizoctonia solani*, post-transcriptional, zinc finger proteins, transcription factor

## Abstract

Transcription factors are key molecules involved in transcriptional and post-transcriptional regulation in plants and play an important regulatory role in resisting biological stress. In this study, we identified a regulatory factor, *OsZF8*, mediating rice response to *Rhizoctonia solani* (*R. solani*) AG1-IA infection. The expression of *OsZF8* affects *R. solani* rice infection. *OsZF8* knockout and overexpressed rice plants were constructed, and the phenotypes of mutant and wild-type (WT) plants showed that *OsZF8* negatively regulated rice resistance to rice sheath blight. However, it was speculated that OsZF8 plays a regulatory role at the protein level. The interacting protein PRB1 of OsZF8 was screened using the yeast two-hybrid and bimolecular fluorescence complementation test. The results showed that OsZF8 effectively inhibited PRB1-induced cell death in tobacco cells, and molecular docking results showed that PRB1 had a strong binding effect with OsZF8. Further, the binding ability of OsZF8-PRB1 to ergosterol was significantly reduced when compared with the PRB1 protein. These findings provide new insights into elucidating the mechanism of rice resistance to rice sheath blight.

## 1. Introduction

Rice sheath blight is a phytopathogenic fungus disease that occurs during rice production and has become the primary disease in some rice-producing regions [1]. It is caused by the necrotrophic pathogen *Rhizoctonia solani* Kühn with a wide host range [2]. Therefore, the research on rice sheath blight is limited by the abundance of pathogen groups, unique classification systems, diverse pathogenic mechanisms, complex interaction regulation, lack of effective resistant varieties, and genetic transformation technology [3]. In recent years, studies on kinases, transcription factors, hormone signals, and synthetic regulatory pathways involved in the interaction between rice and *Rhizoctonia solani* have further revealed the evolution of pathogens, host selection strategies, and interaction regulatory mechanisms [4].

Transcription factors are key molecules in signal transduction and transcriptional regulation in plants. WRKY, MYB, ABI1/B3, NAC, and other transcription factor families regulate rice resistance to *R. solani* [5]. Zinc finger proteins (ZFPs) are a class of transcription factors (TFS) that self-fold by binding to Zn^2+^ to form short and stable finger-like structures [6]. ZFPs are widely distributed in eukaryotic genomes and play important roles in physiological and biochemical processes, such as plant hormone signal transduction, DNA recognition, RNA binding, transcription regulation, growth and development, and biotic and abiotic stress [7,8,9]. Mittler et al. [10] found that the mutation of Zat10 on the C2H2 zinc finger protein of Arabidopsis thaliana enhanced plant tolerance to abiotic stress. Huang et al. [11] identified stress-related zinc finger protein genes in rice and found that ZFP245 plays a role in rice response to salt and drought stress. These results suggest that ZFP245 might promote the tolerance of rice to cold and drought by regulating the proline level and reactive oxygen species (ROS) scavenging capacity. Luo et al. [12] found that SlZF3 regulates plant height by directly inhibiting genes in the gibberellin biosynthesis pathway.

Gene expression is a complex process that contains many steps: RNA transcription, protein translation, and protein stability. Most of the current research on gene expression regulation has focused on transcriptional regulation [13]. For instance, according to a study on maize, ncRNA GARR-2 can interact with ZmUPL1; ZmUPL1 is a kind of enzyme participating in ubiquitin-mediated protein degradation. It is proposed that such interaction will influence the activity of the enzyme, which affects its ability to degrade the downstream targets [14].

The regulation of protein interaction is an advanced view in studying pathogen and host interaction [15]. During the interaction between rice and pathogens, various proteins in rice interact with each other to promote immune-related pathways and regulate the resistance of rice to sheath blight. Yang [16] found that activator of SWEET2a (AOS2) is secreted by pathogens during infection and interacts with WRKY53 and GT1 in rice to form a transcription complex, activate the expression of SWEETs, and promote the transport of carbohydrate compounds from host cells to the outside so that the pathogen can obtain nutrients. Yuan [17] found that PhyB restrained the BZR1-NAC028-CAD8B pathway to regulate rice resistance to sheath blight. Kim [18] showed that tissue-specific activation of SWEET14 with DOF11 conferred rice resistance to sheath blight. Moreover, Sun [19] found that LPA1 activated PIN1a to improve rice resistance to sheath blight.

Sterols, major components of eukaryotic cell membranes, are synthesized in the endoplasmic reticulum and then transported to the cell membrane or other membrane structures through lipid transporter and vesicle transport pathways [20]. In addition to being important components of the cell membrane and other organelle membranes, sterols play an important role in the integrity of the cell membrane, the activity of membrane binding enzymes, membrane fluidity, cell viability, cell material transport, and signal transduction [21]. Different species contain different sterols [22]; for example, in fungi, cell membrane sterols mainly exist in the form of ergosterols. In some plants, sterols occur mainly in the form of phytosterols [23]. In yeast cells, ERGosterol is synthesized using acetyl-CoA (acetyl-CoA) as a raw material and is formed by 25 Erg (ERGosterol biosynthesis) enzymes [21]. Deleting the key Erg gene prevents the cell from synthesizing ergosterol, causing the cell membrane to rupture and resulting in the death of the fungus [24]. Liu [25] identified the transcription factor FgSR in Fusarium graminearum and found that it is activated by the Hog1-MAPK pathway. When FgSR is highly phosphorylated by Hog1, it enters the nucleus and it regulates SWI/SNF complex-mediated chromosome remodeling, activating ergosterol synthesis, which indicates the pathogenic mechanism of ergosterol in pathogenic fungi. In the present study, a regulatory factor, *OsZF8*, involved in the interaction between rice and *R. solani* AG1-IA, was identified. The mechanism by which OsZF8 mediates the regulatory effect of PRB1 on rice sheath blight resistance was analyzed via yeast two-hybrid (Y2H) assays, instantaneous tobacco expression, and molecular docking. The results of this study provide new insights and directions into the further revealing of the immune mechanism and host interaction regulation.

## 2. Results

### 2.1. Analysis of the Expression Patterns of OsZF8

**Figure 1:**
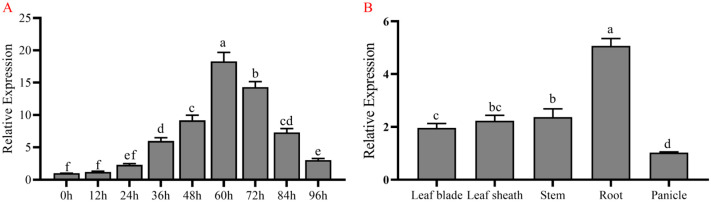
Expression analysis of *OsZF8*. (**A**): Expression analysis of *OsZF8* in response to infection by Y-36 strain. The expression level of *OsZF8* reached its highest 60 h after inoculation. (**B**): Expression analysis of *OsZF8* in different parts of rice. The expression of *OsZF8* was highest in roots and lowest in panicles. Error bars represent means ± SE of three repeated experiments (n = 3). Different letters indicate significant differences (*p* < 0.05).

Based on the expression pattern of *OsZF8* during infection by *R. solani*, the expression of *OsZF8* responded to infection with the Y-36 strain, reaching the highest level 60 h after inoculation. The expression patterns of *OsZF8* in different rice tissues showed that *OsZF8* was expressed in the panicle, stem, leaf, leaf sheath, and root, with the highest expression level in the root and the lowest expression level in the panicle (Figure 1).

### 2.2. Domain and Phylogenetic Analysis of the OsZF8 Protein

**Figure 2:**
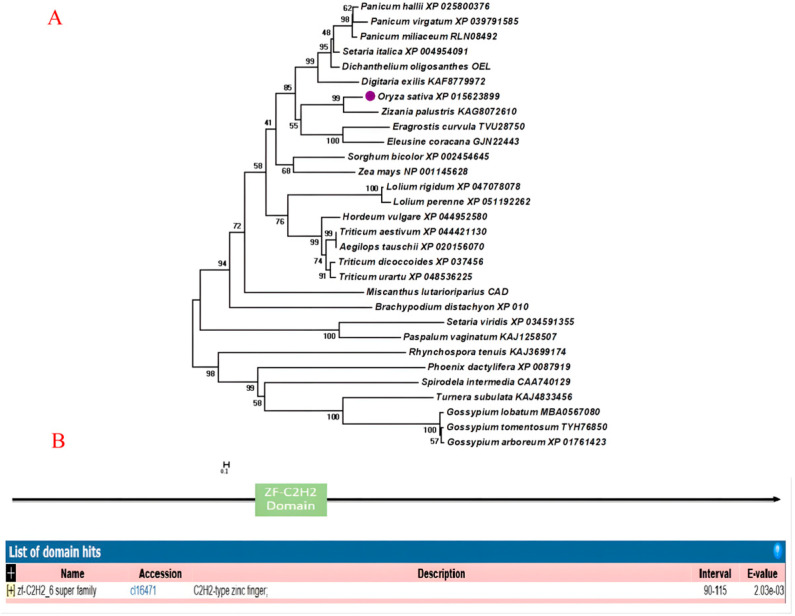
Domain and phylogenetic analysis of the OsZF8. (**A**): Phylogenetic analysis of OsZF8. The numbers indicate the accession numbers of gene sequences of the species in GenBank. (**B**): Protein domain analysis of OsZF8.

The results of the OsZF8 domain and phylogenetic analysis showed that OsZF8 has a typical ZF-C2H2 structure and is characteristic of the OsZF8 transcription factor, which is the rice OsZF8 transcription factor (Figure 2).

### 2.3. Validation of OsZF8 Mutant Plants

**Figure 3:**
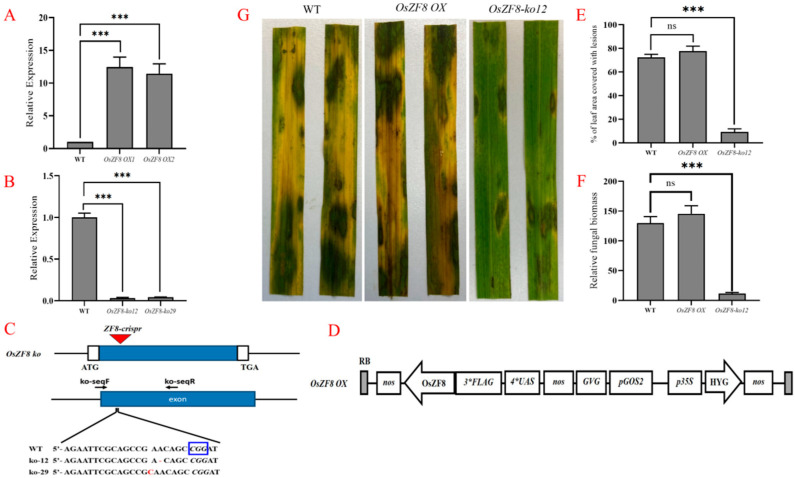
*OsZF8* mutants are more resistant to sheath blight. (**A**): Expression analysis of *OsZF8* in *OsZF8 OX1* and *OsZF8 OX2* plants. (**B**): Expression analysis of *OsZF8* in *OsZF8-ko12* and *OSZF8-ko29* plants. (**C**): Insertion site of *OsZF8* knockout mutant. Blue and white boxes indicate exons and untranslated regions, respectively. The red triangle represents the editing site. (**D**): Construction of *OsZF8* overexpression vector. * represents concatenation of different numbers of tags. (**E**): Relative lesion area on leaves shown in (**G**) leaves inoculated with *R. solani* AG1-IA. (**F**): Relative biomass shown in (**G**) leaves inoculated with *R. solani* AG1-IA. (**G**): The pathogenic symptoms of *OsZF8-ko12* and *OsZF8 OX* plants. ns, no significant difference. *p*-values were analyzed using student’s *t*-test (*p* *** < 0.001).

Sequencing results of the knockout mutant showed that one base A was missing from 107 bp after the initiation site of the *OsZF8*-*ko12* mutant plant, and one base C was inserted into 107BP–108BP of the *OsZF8*-*ko29* mutant plant (Figure 3C). The *OsZF8* expression levels in the obtained *OsZF8*-*ko12*, *OsZF8*-*ko29*, *OsZF8 OX1*, and *OsZF8 OX2* mutants were analyzed. The results showed that the *OsZF8* expression levels in the *OsZF8-ko12* and *OsZF8-ko29* mutant plants were significantly decreased (Figure 3B). *OsZF8* expression was significantly increased in the *OsZF8 OX1* and *OsZF8 OX2* mutant plants (Figure 3A).

### 2.4. Disease Resistance Identification of OsZF8 Mutants

Resistance of the *OsZF8-ko12* and *OsZF8 OX* mutant plants and WT plants to *R. solani* was determined by evaluating the relative lesion area (Figure 3E) and relative biomass (Figure 3F). The results showed that the resistance of the *OsZF8-ko12* mutant plants was significantly enhanced 72 h after inoculation with the Y-36 strain (Figure 3G). The relative lesion area and relative biomass of *OsZF8-ko12* were significantly reduced compared to those of *OsZF8 OX* and WT plants. Similarly, the resistance of the *OsZF8 OX* mutant plants was similar to that of WT plants, suggesting that *OsZF8* negatively regulates rice resistance to rice sheath blight.

### 2.5. Expression Analysis of Defense-Related Genes and Transcriptional Regulatory Genes

**Figure 4:**
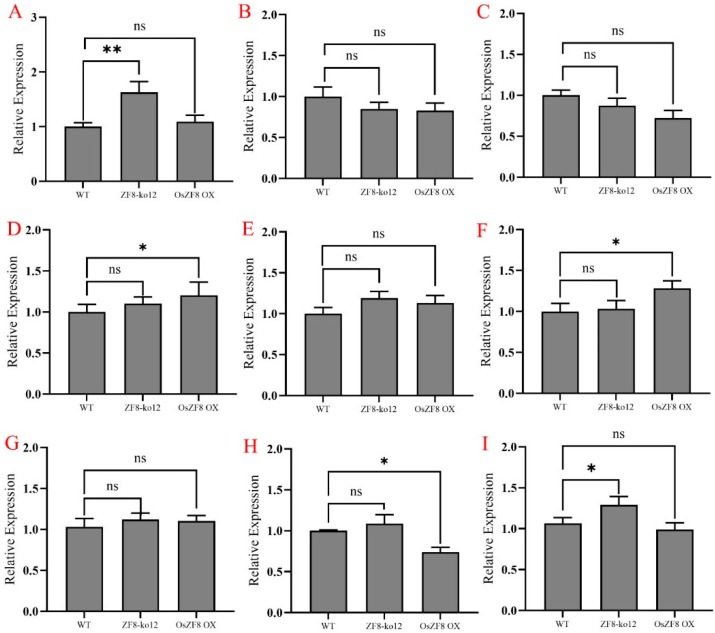
Expression analysis of defense related genes and transcriptional regulatory genes in mutant and wild-type plants. The expression of *NPR1* (**A**), *EIN2* (**B**), *EIL1* (**C**), *D61* (**F**), *D2* (**G**), *GID1* (**H**), and *SLR1* (**I**) in rice plants was examined at 48-h- and *PBZ1* (**D**), *PRb1* (**E**) examined 72-h post inoculation with wild-type and mutants. *p*-values were analyzed using student’s *t*-test (*p* * < 0.05; *p* ** < 0.01). ns, no significant difference.

To analyze the transcriptional regulatory mechanism of OsZF8 in regulating resistance to rice sheath blight, we analyzed changes in expression of the salicylic acid-related resistance gene *NPR1*, the ethylene-related resistance genes *EIN2* and *EIL1*, the brassinolide-related susceptibility genes *D2* and *D61*, the defense response genes *PBZ1* and *PRb1*, and the transcriptional regulatory genes *GID1* and *SLR1* in the mutant and wild-type plants under pathogen stress. The results showed no significant differences in the expression levels of *EIN2*, *EIL1*, *PRb1*, or *D2* between the mutant and wild-type plants. *NPR1* and *SLR1* were significantly upregulated in *OsZF8-ko12*, while *PBZ1* and *D61* were significantly upregulated in *OsZF8 OX*, and *GID1* was significantly downregulated in *OsZF8 OX*. However, the difference was less than 0.5 times. Moreover, the phenotype of the *OsZF8* mutant was inconsistent with the trend in the expression of defense-related genes in response to *R. solani* infection (Figure 4). These results suggest that OsZF8 might not regulate rice resistance to sheath blight at the transcriptional level.

### 2.6. Prb1 Interacts with OsZF8

**Figure 5:**
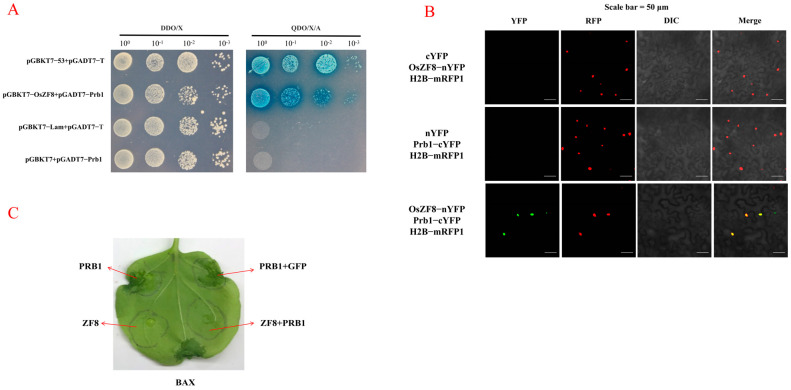
OsZF8 interacts with Prb1. (**A**): Yeast two-hybrid assay for OsZF8 interactions with Prb1. (**B**): Bimolecular fluorescence complementation assay for OsZF8 and Prb1 interaction. pSPYNE-OsZF8, pSPYCE-Prb1 were co-expressed in tobacco leaves. Bar = 50 μm. (**C**): OsZF8 suppresses PRB1-triggered cell death in tobacco. The *Agrobacterium* strain carrying *OsZF8* + *PRB1*, and *gfp* gene construct were co-infiltrated with *Prb1* gene construct into the right leaves, respectively. The *Agrobacterium* strain carrying *Prb1* and *OsZF8* gene construct was infiltrated into the left leaves. Cell death symptoms on the infiltrated leaves were photographed.

Subcellular localization information of OsZF8 was retrieved from the Cell-PLoc website (http://www.csbio.sjtu.edu.cn/bioinf/Cell-PLoc-2, accessed on 1 January 2023), and the results showed that OsZF8 localizes to the nucleus. A total of 60 positive clones were obtained through screening and verification of the rice cDNA yeast library. Furthermore, the interaction between OsZF8 and the Prb1 protein was verified by means of one-to-one yeast two-hybrid assays (Figure 5A). Moreover, the interaction between OsZF8 and Prb1 was verified in tobacco leaf cells by a bimolecular fluorescence complementation test. Fluorescence signals were detected in the nucleus of the co-inoculated sites of pSPYNE-OsZF8 and pSPYCE-Prb1. However, no fluorescence signals were detected in the verification experiment (Figure 5B). Hence, OsZF8 and Prb1 interacted in the plant nucleus.

### 2.7. Effect of OsZF8 on PRB1-Induced Cell Death

As shown in Figure 5C, the Prb1, Prb1 + GFP, and BAX treatments induced cell death in tobacco plants, though no obvious cell death was observed in the ZF8 and ZF8+Prb1 treatment groups. These experimental results indicate that OsZF8 can effectively inhibit Prb1-induced cell death.

### 2.8. Differences in the Ability of the Prb1 Protein to Bind Ergosterol and the OsZF8-Prb1 Protein

**Figure 6:**
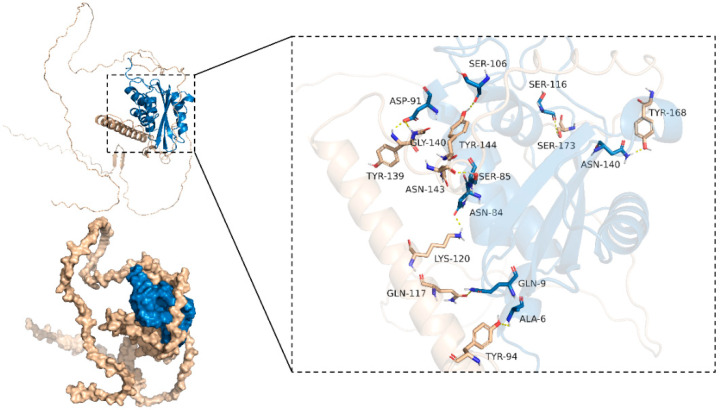
Binding mode of OsZF8 and Prb1 protein. The wheat color in the figure represents the OsZF8 protein model, the blue color represents the Prb1 protein model, and the dashed yellow line represents hydrogen bonding. The following figure uses the same color coding.

In this study, the binding modes of the Prb1 and OsZF8 proteins were determined via ZDOCK 3.0.2 software, and the binding energy of the Prb1 and OsZF8 proteins was determined to be −11.4 kcal/mol. TYR-144, TYR-139, GLY-140, ASN-143, LYS-120, GLN-117, TYR-94, SER-173, and TYR-168 from the OsZF8 protein and SER-106, ASP-91, SER-85, ASN-84, GLN-9, ALA-6, SER-116, and ASN-140 from the PRB1 protein formed hydrogen bonds. These results indicate that Prb1 strongly binds to the ZF8 protein (Figure 6).

**Figure 7:**
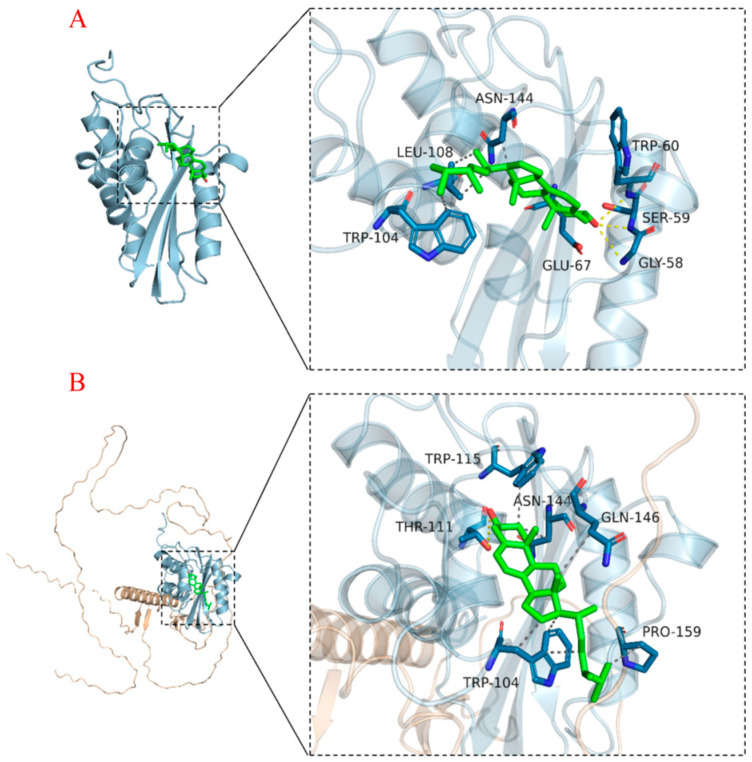
Binding mode of Prb1 protein and OsZF8-Prb1 protein complex with ergosterol (**A**): The binding mode of Prb1 protein and ergosterol. (**B**): Binding mode of OsZF8-Prb1 protein complex and ergosterol. The wheat color in the figure represents the OsZF8 protein model, the blue color represents the Prb1 protein model, and the dashed yellow line represents hydrogen bonding. The following figure uses the same color coding. Ergosterol has a hydrophobic effect on ASN-144, PRO-159, TRP-115, GLN-146, and TRP-104 of the OsZF8-Prb1 protein. This leads to the combination capacity of ergosterol and OsZF8-PRB1 protein being weak.

The binding capacity of the Prb1 protein and the OsZF8-Prb1 protein complex for ergosterol was then analyzed. The binding capacity of the Prb1 protein for ergosterol was −10.3 kcal/mol, and ergosterol could bind to the surface cavity of the Prb1 protein. In addition, ergosterol formed hydrogen bonds with the SER-59, TRP-60, and GLY-58 sites on the Prb1 protein, indicating that ergosterol strongly binds to the Prb1 protein (Figure 7A). The binding energy of the ergosterol and OsZF8-Prb1 protein complex was −8.1 kcal/mol. Ergosterol can bind to the surface cavity of the OsZF8-Prb1 protein complex, but ergosterol can only form hydrogen bonds with THR-111 sites on the OsZF8-Prb1 protein complex. Moreover, the hydrophobic interactions between ergosterol and ASN-144, PRO-159, TRP-115, GLN-146, and TRP-104 of the OsZF8-Prb1 protein complex indicate that the binding effect of ergosterol and the OsZF8-Prb1 protein complex is weak (Figure 7B). These results indicate that the binding of OsZF8 to the Prb1 protein decreases the ability of the Prb1 protein to bind to ergosterol.

## 3. Discussion

The classification system of *Rhizoctonis* spp. is unique. *R. solani* is divided according to the number of nuclei in a single cell into multinucleate, binucleate, and uninucleate. *R. solani* strains are also divided into different anastomosis groups according to whether mycelial fusion occurs. Finally, the strains are divided into different subgroups according to their anastomosis frequency, culture characteristics, host species, pathogenicity, and molecular characteristics. Rice sheath blight and maize sheath blight are caused by the AG1-IA fusion group of *R. solani*, which can lead to severe yield loss [26,27].

Zinc finger proteins are among the largest families of transcription factors in eukaryotes. Zinc finger proteins are divided into 10 types depending on the type of zinc finger motif, C2H2, C2HC, C2HC5, C2C2, CCCH, C3HC4, C4, C4HC3, C6, and C8 [28], of which C2H2 zinc finger proteins are the largest group. Han [29] discovered the first C2H2-type zinc finger protein, EPF1, in petunia and found fourteen EPF1-related genes. Since then, C2H2 zinc finger proteins have been found in many plants, such as Arabidopsis [30], rice [9], and tomato [31], and play important roles in plant growth and development, gene expression and the response to biological and abiotic stresses [32]. Chu et al. [33] conducted a genome-wide identification of ZFP genes in three species of grapevine, and examined the VvZFP family genes involved in response to methyl jasmonate (MeJA), abscisic acid (ABA), and salicylic acid (SA). Through an interaction network between TtC2H2-ZF proteins with identified C2H2-ZF TFs in *A. thaliana*, Faraji (2018) found that, ZFP1, ZFP2, ZFP3, ZFP10, and ZFP11 regulate many down-stream genes involved in multiple biological processes, including stress and hormone responses [34].

Previous studies showed that zinc finger proteins not only have transcriptional regulatory activities but also can interact with other proteins to regulate protein activity, thus regulating plant growth and disease resistance. Rice OsZFP can directly recognize P8 protein of SRBSDV and regulate rice resistance to SRBSDV [35]. OsZFP213 interacts with OsMAPK3 to enhance the ability of scavenging ROS and synergistically regulate the salt tolerance of rice [36].

Post-translational modifications of protein such as ubiquitination, phosphorylation, glycosylation, and polymerization can affect the stability and activity of zinc finger proteins, and thus affect their biological functions. MdBBX7/MdCOL9 can be directly ubiquitinated and degraded by E3 ubiquitin ligase MIEL1, which reduces apple tolerance to drought stress [37]. CCCH-type zinc finger protein OsLIC, as a substrate of OsMAPK6, can be activated by phosphorylation of OsMAPK6 to regulate rice resistance to *Xoo* and *Xoc* [38]. AtZAT6 positively modulates expression levels of stress-related genes by directly binding to the promoter region of pathogen-related genes like PR1 and PR5 [39]. ZAT10 and EIN3 can be phosphorylated by MPK3/6 [40], and ZAT12 is a direct target of EIN3, so that ZAT12 also may be involved in MAPK signal cascades [41].

At present, the function of rice zinc finger proteins, OsZF8, has not been reported. In this study, we identified that the expression of *OsZF8* was related to *R. solani* infection. Further experiments speculated that OsZF8 might regulate rice resistance to *R. solani* not only at the transcriptional level but also through protein regulation. Zinc finger-like transcription factors have protein-regulatory functions in addition to transcriptional regulation; therefore, the regulatory function of OsZF8 was further studied. The OsZF8-interacting protein Prb1 was screened through tests in vitro and in vivo. The Prb1 protein, a downstream functional protein involved in rice defense response to disease, directly inhibits pathogens. We found that ergosterol and the Prb1 protein strongly bind to each other, but ergosterol and the OsZF8-Prb1 protein complex only weakly bind. In summary, OsZF8 inhibits the binding of the Prb1 protein to ergosterol by binding the Prb1 protein and negatively regulates rice resistance to *R. solani*. Based on the above results, it is speculated that although some fungi can synthesize ergosterol by themselves, the binding efficiency of PRB-1 to ergosterol is higher than its autosynthesis rate, and thus has a suppression on fungi.

## 4. Materials and Methods

### 4.1. Plant Growth and R. solani Inoculation

*R. solani* AG1-IA (Y-36), Y2H strains, Y187 strains, DH5α, EHA105 strains, and GV3101 strains were preserved at the College of Plant Protection, Shenyang Agricultural University (Shenyang, Liaoning, China). All of the rice plants treated with *R. solani* were cultured in the Shenyang Agriculture University greenhouse at 23–30 °C, 80% relative humidity (RH), and 12-h light/12-h dark photoperiod. Tobacco was cultured at 25 °C under 16 h of light and at 23 °C under 8 h of darkness. The *OsZF8* knockout mutant *OsZF8-ko12* and the overexpressing mutant *OsZF8 OX* were planted in the greenhouse of Shenyang Agricultural University. The *R. solani* strain AG1-IA was cultured on solid PDA (potato dextrose agar) medium at 28 °C in an incubator. The inoculation method referred to the study by Cao et al. [42]. The rice cDNA library plasmid, pGBKT7 vector, pGADT7 vector, pSPYNE vector, pSPYCE vector, pINDEX2 vector, and pCBDEST vector were all sourced from the College of Plant Protection, Shenyang Agricultural University.

*OsZF8-ko12* plants and *OsZF8 OX* plants were grown for 60 days; WT plants were grown for 90 days. The roots, stems, leaves, leaf sheaths, and ears of the WT plants were collected as samples for the analysis of *OsZF8* expression in different rice organs (OsZF8F/OsZF8R). The Y-36 strain was inoculated onto isolated leaves of inverted two-leaf plants (60 days), and samples were collected at 0 h, 12 h, 24 h, 36 h, 48 h, 60 h, 72 h, 84 h, and 96 h for analysis of the expression of *OsZF8* in response to infection by Y-36. *OsZF8-ko12* and *OsZF8 OX* plants were used as mutant plants for verification. The expressions of *NPR1*, *EIN2*, *EIL1*, *D61*, *D2*, *GID1*, and *SLR1* in WT, *OsZF8-ko12*, and *OsZF8 OX* plants were analyzed 48 h after inoculation with the Y-36 strain. The primers used were as follows: qNPR1F/qNPR1R, qEIN2F/qEIN2R, qEIL1F/qEIL1R, qD61F/qD61R, qD2F/qD2R, qGID1F/qGID1R, and qSLR1F/qSLR1R. The expressions of *PBZ1* and *PR1b* in WT, *OSZF8-ko12*, and *OsZF8 OX* plants were analyzed 72 h after inoculation with the Y-36 strain. The primers were PBZ1F/PBZ1R and PR1BF/PR1BR. In all experiments involving *OsZF8 OX* plants, 20 μM dexamethasone (DEX) was used to spray 24 h before the experiment. All primers are shown in Table S1.

### 4.2. Construction of OsZF8 Mutant Plants

The *OsZF8* knockout mutant—clustered regularly interspaced short palindromic repeats/CRISPR-associated protein 9 (CRISPR/Cas9)—was used to construct the OsZF8 knockout construct; the target sequence was designed, and BGE Gene Technology Co., Ltd. (Changzhou, Jiangsu, China) constructed the knockout mutant. With the use of hygromycin screening and sequencing, the transgene was verified, and a positive T0 generation knockout mutant was obtained. The primers used were SpeI-ZF8F/SpeI-ZF8R. After the single T0 knockout mutant plant was propagated, the T1 knockout plant was planted, and sequencing and qPCR verification were performed again. The primers used were ZF8qF/ZF8qR.

OsZF8 overexpression mutant: Rice cDNA was used as the template for the amplification of *OsZF8*, and after the amplified fragment was recovered, the enzyme digestion product was ligated to the pINDEX2-FLAG vector via SpeI and XhoI sites. The recombinant protein was subsequently transformed into DH5α cells, after which the plasmid was extracted for sequencing. The primers used were ZF8-qF/M13R. The correct overexpression vector was sequenced and transformed into the EHA105 strain. Positive clones were selected, cultured, and subsequently sent to Baige Gene Technology Co., Ltd.(Changzhou, Jiangsu, China) for overexpressing plant construction. Positive T0 mutant plants were obtained via hygromycin screening. The primers used were ZF8-qF/M13R. Transformed plants with the correct target band size were selected for breeding, and the expression level of ZF8 in T1 generation plants was detected by qPCR. The primers used were ZF8qF/ZF8qR.

### 4.3. RNA Extraction and Quantitative Real-Time (qRT)-PCR Analysis

Briefly, 0.1 g of inoculated sample was fully ground with liquid nitrogen for RNA extraction and cDNA synthesis according to the instructions for the Ultrapure RNA Kit Extraction Kit (CWBIO, CW0581, Taizhou, Jiangsu, China) and HiScript III RT SuperMix for qPCR Kit (Vazyme, R323-01, Nanjing, Jiangsu, China). ChamQ Universal SYBR qPCR Master Mix (Vazyme, Q711) was used to configure the reaction system, and real-time fluorescent quantitative PCR was performed using a CFX Duet fluorescent quantitative PCR instrument (Bio-Rad, Hercules, CA, USA). Two technical and three biological replicates were used for each analysis. The fluorescence signal was collected after the annealing/extension step. Ct values were determined after the reaction, and the data were analyzed using the 2^−ΔΔCt^ method [43].

### 4.4. Yeast Two-Hybrid Assay

A cDNA library of rice was screened with pGBKT7-OsZF8 as bait and via the mating method. The CDS of the interacting protein PRB1 was obtained, rice cDNA was used as the template, the target protein CDS was cloned, and the sequence was inserted into pGADT7 via enzyme digestion. After enzyme digestion and sequencing verification, the AD and BD plasmids were transferred to the Y2H strain, which was subsequently cultured on SD/-Trp/-Leu plates. Positive colonies were selected and diluted according to the gradient, and SD/-Trp/-Leu/-His/-Ade/X-alpha-gal/AbA was used for detection. Primers (ADF/ADR, AD-PRB1F/AD-PRB1R) are shown in Table S1.

### 4.5. Bimolecular Fluorescence Complementation Assay

The pSPYNE-OsZF8 plasmid and pSPYCE-AD plasmid were constructed via homologous recombination and subsequently transferred to Agrobacterium EHA105. PCR was used to amplify the NbH2B fragment harboring the attB1 and attB2 sequences at both ends (the stop codon was removed). The NbH2B gene fragment was subsequently transferred into the pH7RWG2.0 vector. Strains carrying pSPYNE-OsZF8, pSPYCE-AD, and pH7RWG2.0-H2B were infiltrated and inoculated onto tobacco leaves for 30 days, after which fluorescence was observed under a 488–550 mm laser confocal microscope after 48 h.

### 4.6. Instantaneous Expression of Tobacco

The protein-coding sequences of OsZF8 and Prb1 were reconstructed to the pCBDEST-transient sequence using the Gateway method; GFP and BAX were used as negative and positive controls, respectively. The obtained plasmid was subjected to thermal shock transformation using the competent cell line GV3101, which was subsequently coated on a plate containing LB + Rif + Kana for positive cloning screening. The positive clone was added to 2 mL of liquid LB (containing Rif + Kana) and shaken at 28 °C and 220 r·min^−1^ for 16 h, and the culture was diluted to a final concentration of 0.5 optical density (OD). Tobacco cultured for 30 days was used in the test. The penetration solution was gently injected into tobacco cells from the back of the leaves using a 1 mL sterile syringe. The penetration diameter of each injection site was approximately 1.5 cm. The surface of the leaves was wiped dry. After 3 days of culture, the degree of cell death was observed and photographed [44,45].

### 4.7. Molecular Docking

ASphafold 2 software was used to predict the crystal structure of the Prb1 and OsZF8 proteins. PyMol 2.5.3 software removed water molecules, hydrogen atoms, and nontarget structural proteins from the PRB1 and OsZF8 proteins. ZDOCK 3.0.2 software was employed to simulate the binding mode of PRB1 and OsZF8. After docking, AMBER 18 software was used to minimize the energy under the ff14SB force field, and Prodigy software (v1.12.0) was used to evaluate the binding energy. PyMol 2.5.4 software was used for visual analysis based on the optimal binding energy binding mode [46].

Prb1 and OsZF8-Prb1 protein complexes were derived from the above prediction and bonding results. The 3D structure of ergosterol (CAS: 57-87-4) was downloaded from the PUBCHEM database and minimized under the MMFF94 force field. AutoDock Vina 1.1.2 software was used to dock proteins with small molecules, and PyMol 2.5 software was used to analyze the docking results visually [47,48].

### 4.8. Statistical Analyses

The structure and functional domains of the OsZF8 protein were analyzed via NCBI-SMART online software (https://blast.ncbi.nlm.nih.gov/smartblast/?LINK_LOC=BlastHomeLink, accessed on 24 May 2024), and a phylogenetic tree of the OsZF8 protein was constructed via MEGA5.0 software. Statistical analyses were performed using Microsoft Office Excel 2016, and significance analysis was performed using SPSS 22.0. Also, Student’s *t*-test was used to compare the differences between the two groups. Differences between the groups were considered significant with at least *p* < 0.05.

## 5. Conclusions

In summary, OsZF8 was significantly up-regulated after *R. solani* AG1-IA inoculation, suggesting that OsZF8 plays an important regulatory role in the interaction between rice and *Rhizoctonia solani*. In this study, OsZF8 expression pattern, mutant phenotype identification, bioinformatics, and expression of related defense genes were analyzed. Transient expression, yeast two-hybrid, and molecular docking were used to analyze the regulatory function of OsZF8 at post-transcriptional protein level. OsZF8 inhibits the binding ability of Prb1 protein to ergosterol by binding to Prb1 protein, and negatively regulates rice resistance to sheath blight. This results provided new insights for understanding the mechanism of resistance to rice sheath blight.

## Data Availability

Data are contained within the article.

## Supplementary Materials

The following supporting information can be downloaded at: https://www.mdpi.com/article/10.3390/ijms25115787/s1.

## References

[1] Molla K.A., Karmakar S., Molla J., Bajaj P., Varshney R.K., Datta S.K., Datta K. (2020). Understanding sheath blight resistance in rice: The roadbehind and the road ahead. Plant Biotechnol. J..

[2] Li D., Li S., Wei S.H., Sun W.X. (2021). Strategies to Manage Rice Sheath Blight: Lessons from Interactions between Rice and *Rhizoctonia solani*. Rice.

[3] Singh P., Mazumdar P., Harikrishna J.A., Babu S. (2019). Sheath blight of rice: A review and identification of priorities for future research. Planta.

[4] Liang X.X., Zhou J.M. (2018). Receptor-like cytoplasmic kinases: Central players in plant receptor kinase-mediated signaling. Annu. Rev. Plant Biol..

[5] Rushton D.L., Tripathi P., Rabara R.C., Lin J., Ringler P., Boken A.K., Langum T.J., Smidt L., Boomsma D.D., Emme N.J. (2012). WRKY transcription factors: Key components in abscisic acid signalling. Plant Biotechnol. J..

[6] Agnieszka K.M. (2012). Involvement of plant C2H2-type zinc finger transcription factors in stress responses. Plant Sci..

[7] Zhang A., Liu D., Hua C., Yan A., Liu B., Wu M., Liu Y., Huang L., Ali I., Gan Y. (2016). The Arabidopsis gene zinc finger protein 3(ZFP3) is involved in salt stress and osmotic stress response. PLoS ONE.

[8] Shuai Y., Feng G., Yang Z., Liu Q., Han J., Xu X., Nie G., Huang L., Zhang X. (2022). Genome-wide identification of C2H2-type zinc finger gene family members and their expression during abiotic stress responses in orchardgrass (*Dactylis glomerata*). Genome.

[9] Pinky A., Rita A., Swatismita R., Ashok K.S., Vijay P.S., Hiroshi T., Tyagi A.K. (2007). Genome-wide identification of C2H2 zinc-finger gene family in rice and their phylogeny and expression analysis. Plant Mol. Biol..

[10] Mittler R., Kim Y., Song L., Coutu J., Coutu A., Yilmaz S.C., Zhu J.K. (2006). Gain-and loss-of-function mutations in Zat10 enhance the tolerance of plants to abiotic stress. Febs Lett..

[11] Huang J., Sun S.J., Xu D.Q., Yang X., Bao Y.M., Wang Z.F., Tang H.J., Zhang H. (2009). Increased tolerance of rice to cold, drought and oxidative stresses mediated by the overexpression of a gene that encodes the zinc finger protein ZFP245. Biochem. Biophys. Res. Commun..

[12] Luo J., Tang Y., Chu Z., Peng Y., Chen J., Yu H., Shi C., Jafar J., Chen R., Tang Y. (2023). SlZF3 regulates tomato plant height by directly repressing SlGA20ox4 in the gibberellic acid biosynthesis pathway. Hortic. Res..

[13] Kan Q., Li Q. (2023). Post-transcriptional and translational regulation of plant gene expression by transposons. Curr. Opin. Plant Biol..

[14] Li W., Chen Y., Wang Y., Zhao J., Wang Y. (2022). Gypsy retrotransposon-derived maize lncRNA GARR2 modulatesgibberellin response. Plant J..

[15] Zhu X.F., Yuan D.P., Zhang C., Li T.Y., Xuan Y.H. (2018). RAVL1, an upstream component of brassinosteroid signalling and biosynthesis, regulates ethylene signalling via activation of EIL1 in rice. Plant Biotechnol. J..

[16] Yang S., Fu Y., Zhang Y., Yuan D.P., Li S., Kumar V., Mei Q., Xuan Y.H. (2022). Rhizoctonia solani transcriptional activator interacts with rice WRKY53 and grassy tiller 1 to activate SWEET transporters for nutrition. J. Aev. Res..

[17] Yuan D.P., Yang S., Feng L., Chu J., Dong H., Sun J., Chen H., Li Z., Yamamoto N., Zheng A. (2022). Red-light receptor phytochrome B inhibits BZR1-NAC028-CAD8B signaling to negatively regulate rice resistance to sheath blight. Plant Cell Environ..

[18] Kim P., Xue C.Y., Song H.D., Gao Y., Feng L., Li Y., Xuan Y.H. (2021). Tissue specific activation of DOF11 promotes rice resistance to sheath blight disease and increases grain weight via activation of SWEET14. Plant Biotechnol. J..

[19] Sun Q., Li T.Y., Li D.D., Wang Z.Y., Li S., Li D.P., Xuan Y.H. (2019). Overexpression of Loose Plant Architecture 1 increases planting density and resistance to sheath blight disease via activation of PIN-FORMED 1a in rice. Plant Biotechnol. J..

[20] Mayor S., Sabharanjak S., Maxfield F.R. (1998). Cholesterol-dependent retention of GPI-anchored proteins in endosomes. EMBO J..

[21] Sokolov S.S., Trushina N.I., Severin F.F., Knorre D.A. (2019). Ergosterol Turnover in Yeast: An Interplay between Biosynthesis and Transport. Biochemistry.

[22] Gimpl G., Wiegand V., Burger K., Fahrenholz F. (2002). Cholesterol and steroid hormones: Modulators of oxytocin receptor function. Prog. Brain Res..

[23] Desmond E., Gribaldo S. (2009). Phylogenomics of sterol synthesis: Insights into the origin, evolution, and diversity of a key eukaryotic feature. Genome Biol. Evol..

[24] Yan X., Ma W.B., Li Y., Wang H., Que Y.W., Ma Z.H., Talbot N.J., Wang Z.Y. (2011). A sterol 14alpha demethylase is required for conidiation, virulence and for mediating sensitivity to sterol demethylation inhibitors by the rice blast fungus *Magnaporthe oryzae*. Fungal Genet. Biol..

[25] Liu Z., Jian Y., Chen Y., Kistler H.C., He P., Ma Z., Yin Y. (2019). A phosphorylated transcription factor regulates sterol biosynthesis in Fusarium graminearum. Nat. Commun..

[26] Chen B., Han H., Hou J., Bao F., Tan H., Lou X., Wang G., Zhao F. (2022). Control of maize sheath blight and elicit induced systemic resistance using Paenibacillus polymyxa strain SF05. Microorganisms.

[27] Senapati M., Tiwari A., Sharma N., Chandra P., Bashyal B.M., Ellur R.K., Bhowmick P.K., Bollinedi H., Vinod K.K., Singh A.K. (2022). *Rhizoctonia solani* Kühn pathophysiology: Status and prospects of sheath blight disease management in rice. Front. Plant Sci..

[28] Cui H., Chen J., Liu M., Zhang H., Zhang S., Liu D., Chen S. (2022). Genome-wide analysis of C2H2 zinc-finger gene family and its response to cold and drought stress in sorghum [*Sorghum bicolor* (L.) Moench]. Int. J. Mol. Sci..

[29] Han G., Lu C., Guo J., Sui N., Wang B. (2020). C2H2 zinc-finger proteins: Master regulators of abiotic stress responses in plants. Front. Plant Sci..

[30] Englbrecht C.C., Schoof H., Böhm S. (2004). Conservation, diversification and expansion of C2H2 zinc-finger proteins in the Arabidopsis thaliana genome. BMC Genom..

[31] Weng L., Zhao F., Li R., Xu C., Chen K., Xiao H. (2015). The zinc-finger transcription factor SlZFP2 negatively regulates abscisic acid biosynthesis and fruit ripening in tomato. Plant Physiol. Bethesda.

[32] Liu Y., Khan A.R., Gan Y. (2022). C2H2 Zinc Finger Proteins Response to Abiotic Stress in Plants. Int. J. Mol. Sci..

[33] Chu M., Wang T., Li W., Liu Y., Bian Z., Mao J., Chen B. (2023). Genome-Wide Identification and Analysis of the Genes Encoding Q-Type C2H2 Zinc Finger Proteins in Grapevine. Int. J. Mol. Sci..

[34] Faraji S., Rasouli S.H., Kazemitabar S.K. (2018). Genome-wide exploration of C2H2 zinc finger family in durum wheat (*Triticum turgidum* ssp. Durum): Insights into the roles in biological processes especially stress response. Biometals.

[35] Li J., Cai N.J., Xue J., Yang J., Chen J.P., Zhang H.M. (2017). Interaction between southern rice black-streaked dwarf virus minor core protein P8 and a rice zinc finger transcription factor. Arch. Virol..

[36] Zhang Z., Liu H., Sun C., Ma Q., Bu H., Chong K., Xu Y. (2018). A C2H2 zinc-finger protein OsZFP213 interacts with OsMAPK3 to enhance salt tolerance in rice. J. Plant Physiol..

[37] Chen P., Zhi F., Li X., Shen W., Yan M., He J., Bao C., Fan T., Zhou S., Ma F. (2022). Zinc-finger protein MdBBX7/MdCOL9, a target of MdMIEL1 E3 ligase, confers drought tolerance in apple. Plant Physiol..

[38] Wang L.H., Chen J., Zhao Y.Q., Wang S.P., Yuan M. (2022). OsMAPK6 phosphorylates a zinc finger protein OsLIC to promote downstream OsWRKY30 for rice resistance to bacterial blight and leaf streak. J. Integr. Plant Biol..

[39] Shi H., Wang X., Ye T., Chen F., Deng J., Yang P., Zhang Y., Chan Z. (2014). The Cysteine2/Histidine2-Type Transcription Factor Zinc finger of arabidopsis thaliana6 modulates biotic and abiotic stress responses by activating salicylic acid-related genes and c-repeat-binding factor Genes in Arabidopsis. Plant. Physiol..

[40] Yoo S.D., Cho Y.H., Tena G., Xiong Y., Sheen J. (2008). Dual control of nuclear EIN3 by bifurcate MAPK cascades in C2H4 signalling. Nature.

[41] Xie M., Sun J., Gong D., Kong Y. (2019). The Roles of Arabidopsis C1-2i Subclass of C2H2-type Zinc-Finger Transcription Factors. Genes.

[42] Cao W., Zhang H., Zhou Y., Zhao J., Lu S., Wang X., Zuo S. (2021). Suppressing chlorophyll degradation by silencing OsNYC3 improves riceresistance to Rhizoctonia solani, the causal agent of sheath blight. Plant Biotechnol. J..

[43] Livak K.J., Schmittgen T.D. (2013). Analysis of relative gene expression data using real-time quantitative PCR and the 2(-delta delta C(T)) method. Methods.

[44] Wang Q., Han C., Ferreira A.O., Yu X., Ye W., Tripathy S., Kale S.D., Gu B., Sheng Y., Sui Y. (2011). Transcriptional programming and functional interactions within the Phytophthora sojae RXLR effector repertoire. Plant Cell.

[45] Chang Q.L., Xu H.J., Peng Y.L., Fan J. (2019). Subtractive hybridization-assisted screening and characterization of genes involved in the rice-*Magnaporthe oryzae* interaction. Phytopathol. Res..

[46] Pierce B.G., Hourai Y., Weng Z. (2011). Accelerating protein docking in ZDOCK using an advanced 3D convolution library. PLoS ONE.

[47] Trott O., Olson A.J. (2010). Software news and update AutoDock Vina: Improving the speed and accuracy of docking with a new scoring function, efficient optimization, and multithreading. J. Comput. Chem..

[48] Ravindranath P.A., Forli S., Goodsell D.S., Olson A.J., Sanner M.F. (2015). AutoDockFR: Advances in protein-ligand docking with explicitly specified binding site flexibility. PLoS Comput. Biol..

