# Supplementary material for: Mechanism Analysis of OsZF8-Mediated Regulation of Rice Resistance to Sheath Blight
Source: Int J Mol Sci. 2024 May 26;25(11):5787. doi: 10.3390/ijms25115787 (PMC11171851; doi:10.3390/ijms25115787)
Supplement: Supplementary file 1 [file ijms-25-05787-s001.zip › Table S1.pdf]

Table S1 Primers used in this study

Function	Gene name	Primer sequence
qRT-PCR	OsZF8F	GTTTTGTGTCATTGTGTGTG
	OsZF8R	CACACACAATGACACAAAAC
	qNPR1F	GCGGAGAAGAGGAAGAGGTT
	qNPR1R	AACCTCTTCCTCTTCTCCGC
	qEIN2F	ATCTGCCTTTGCTGTTGGTG
	qEIN2R	CACCAACAGCAAAGGCAGAT
	qEIL1F	CACAATGTCCAGTGCCCGCATAG
	qEIL1R	CTCTCCATGATCGTGGCATTGTC
	qD61F	GATTCCTTGTGGGCAGCGAT
	qD61R	ATCGCTGCCCACAAGGAATC
	qD2F	ATGTGATAACAGAGACGCTGCGGT
	qD2R	TGGTGACCAAGTGGTGAAGGAAGA
	qGID1F	TACAACATTCTGCGGCGGGC
	qGID1R	GCCCGCCGCAGAATGTTGTA
	qSLR1F	GCAAGGACAAGGTGATGGCG
	qSLR1R	CGCCATCACCTTGTCCTTGC
	PBZ1F	CCCTGCCGAATACGCCTAA
	PBZ1R	CTCAAACGCCACGAGAATTTG
	PR1BF	GCGTCTTCATCACATGCAACTA
	PR1BR	ACCTGAAACAGAAAGAAACAGAGG
Gene overexpression vector	SpeI-ZF8F	TTactagtATGGCGAAGCCGCAGGACAT
	SpeI-ZF8R	TTctcgagCAAATGGAGGTCCAAGCTGACACC
	ZF8qF	GAGATGGTGCCGATGAGCTT
	ZF8qR	GCAGTAGTGGCACTCGAACT
	M13R	ATGGAGGTCCAAGCTGACAC
	ADF	CTATTCGATGATGAAGATACCCCACCAAACCCAAAAAAAGAG
	ADR	GTGAACTTGCGGGGTTTTTCAGTATCTACGATT

Y2H	AD-PRB1F	CAGAACTCGCCGCAGGACTT
	AD-PRB1R	AAGTCCTGCGGCGAGTTCTG
BiFC	Pbr1-pSPYCE-BamHI-F	GCCACTAGTGGATCCATGGCGACCACGCCGGCGGT
	Pbr1-pSPYCE-XhoI-F	AGCGGTACCCTCGAGAGCAGAGAGAGTGAGGCCAC
	ZF8-pSPYNE-BamHI-F	GCCACTAGTGGATCCAGCTTGGTAGTCCCCCTCTG
	ZF8-pSPYNE-XhoI-F	AGCGGTACCCTCGAGTCTTTTGCCGTTTTGGAATTG
Construction of transient expression vector	ZF8-attb-F	AAAAAGCAGGCTATGGCGAAGCCGCAGGACAT
	ZF8-attb-R	AGAAAGCTGGGTTTACAAATGGAGGTCCAAGCTGACACC
	RPB1-attb-F	AAAAAGCAGGCTATGGAGACACCAAAGATATCGGGAG
	RPB1-attb-R	AGAAAGCTGGGTTTAAGCAGAGAGAGTGAGGCCAC
	attb-GFP-F	AAAAAGCAGGCTATGAAGACTAATCTTTTTCTCTTTC
	attb-GFP-R	AGAAAGCTGGGTTTAGAGTTCGTCGTGTTTGTATAG
